# Dual Comb Spectrometer for the Determination of Stable Isotopic Ratios of Atmospheric CO_2_ with Sub-Permille Precision at Atmospheric Pressure

**DOI:** 10.1021/acs.analchem.5c04845

**Published:** 2025-12-16

**Authors:** Jens Goldschmidt, Nicolas Brugger, Leonard Nitzsche, Ponkanok Nitzsche, Cem Dinc, Christian Weber, Ingo Breunig, Katrin Schmitt, Frank Kühnemann, Jürgen Wöllenstein

**Affiliations:** † Laboratory for Gassensors, Department of Microsystems Engineering−IMTEK, University of Freiburg, Georges-Köhler-Allee 102, 79110 Freiburg, Germany; ‡ Fraunhofer Institute for Physical Measurement Techniques−IPM, Georges-Köhler-Allee 301, 79110 Freiburg, Germany; § Laboratory for Optical Systems, Department of Microsystems Engineering−IMTEK, University of Freiburg, Georges-Köhler-Allee 102, 79110 Freiburg, Germany; ∥ Institute of Physics, University of Freiburg, Hermann-Herder-Straße 3, 79104 Freiburg, Germany

## Abstract

We present a mid-infrared dual comb spectrometer for the precise determination of the isotopic ratio of the stable CO_2_ isotoplogues ^12^C^16^O_2_ and ^13^C^16^O_2_ under atmospheric pressure. The spectrometer is based on electro-optic intensity modulation at 1550 nm wavelength and subsequent wavelength flexible conversion to the mid-infrared. Here, the fundamental absorptions of CO_2_ in the ν_3_ band around 4.3 μm wavelength (2300 cm^–1^) were accessed to achieve the needed sensitivity to investigate the isotopic composition at atmospheric concentrations. The high spectral resolution of 0.004 cm^–1^ and spectral coverage of 8 cm^–1^ enable the measurements of the three most abundant CO_2_ isotopologues ^12^C^16^O_2_, ^13^C^16^O_2_ and ^16^O^12^C^18^O at ambient pressure. The high average signal-to-noise ratio per comb mode of 51 dB and a noise equivalent absorption coefficient of 5.4(9)·10^–6^ cm^–1^ Hz^–1/2^ ensures high precision. After an integration time of 172 s a precision on the stable isotopic ratio (δ^13^C) of <0.1‰ is achieved according to Allan deviation analysis. A linearity analysis on the measured concentrations of the single isotopologues results in coefficients of determination (*R* ^2^) of 0.999 for CO_2_ concentrations ranging from 300 to 450 ppm, whereas for the measurement of δ^13^C-values ranging from −36.5‰ and −5.4‰ a coefficient of determination of 0.998 was achieved. This linear behavior and the high precision on the measurements demonstrate the great potential of the presented dual comb spectrometer for atmospheric research. Especially, where measurements under low pressure must be avoided, the here presented system is a promising alternative to established quantum cascade laser-based systems.

## Graphical abstract

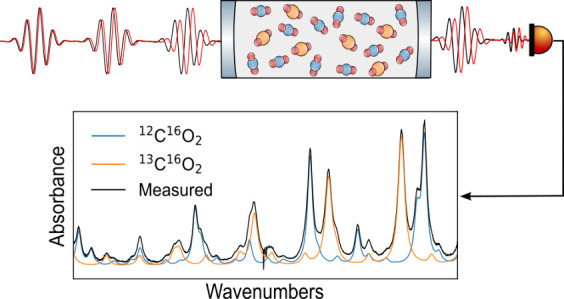

The determination of stable isotopic compositions of CO_2_ in the atmosphere is required for understanding the global carbon cycle and the anthropogenic impact on the climate. The stable isotopic ratio in plants and soil can indicate ecological changes in the environment and provides insights on the exchange of trace gases between the ecosystem and the atmosphere. This results in distinct isotopic fingerprints, which gives information on different processes of the plants′ metabolism and the carbon transfer between soil and plants.^1–6^ A particular relevant tool is the measurement of the composition of the stable isotopologues ^12^C^16^O_2_ and ^13^C^16^O_2_ (δ^13^C). This task is particularly demanding as both isotopologues have to be measured in parallel with high sensitivity. Cross sensitivities between the target gases and to other gases must be avoided, to achieve the demanded precision. This often leads to measurements under reduced gas pressure. However, some measurement scenarios like the investigation of the stable isotopic ratio in soil require measurements under ambient pressure, because it is necessary to avoid pumping and to put the measurement cell into the ground so the target gas can diffuse into the cell.^7^ This sets strict requirements for the analyzing instrument especially when taking field deployment into account.

The current gold standard in analyzing the stable isotopic ratio is isotope ratio mass spectrometry (IRMS). This tool offers very high precision and accuracy but requires extensive sample preparation and does not allow field deployment due to its high complexity.^8,9^ Optical techniques, particularly tunable laser absorption spectroscopy^10–14^ and cavity ring down spectroscopy,^15–18^ which use narrow line width tunable lasers, allow for high sensitivity and acquisition rates. A precision on the δ^13^C-value of <0.1‰ can be reached within seconds of integration time. In contrast to IRMS no extensive sample preparation is needed and in situ measurements, where the sample can be recollected, are possible. They also enable the analysis of species with equal molecular mass. However, due to the small spectral coverage of tunable lasers, measurements are prone to cross sensitivities due to overlapping absorption profiles. So special care has to be taken in the choice of the spectral area and measurements have to be performed under low pressure to minimize the overlap of absorptions. This imposes a limitation as with decreasing pressure the absorption strength also decreases resulting in reduced sensitivity. Alternatively, to further minimize cross sensitivities the spectral coverage can be increased, e.g. with Fourier Transform Infrared Spectroscopy (FTIR).^19^ However, FTIR systems come with inherent drawbacks. The inherent instrument function of these systems limits the achievable spectral resolution and so cause a subtle source of cross sensitivity. To reach the desired precision, integration times of several minutes are needed to reach a precision of 0.1‰ due to the use of incoherent light sources. In combination with moving parts these systems are less suited for field deployment.

Dual frequency comb spectroscopy (DCS) based on electro-optic intensity modulation fills the gap between tunable laser and FTIR instruments.^20,21^ The generation of the dual comb with electro-optic intensity modulation offers a high signal-to-noise ratio per comb mode compared to other frequency comb techniques by sacrificing spectral coverage. This allows for short integration times with a moderate spectral span in the range of tens of wavenumbers and enables measurements under atmospheric pressure with reduced cross sensitivities. Dual comb spectrometers in the near-infrared (NIR) are well-known and established systems.^22^ However, because of the weak overtone transitions of small molecules in the NIR, measurements of the stable isotopic ratio of CO_2_ in atmospheric conditions are not possible to perform without the use of cavities to extend the absorption path length. This drastically increases system complexity.^23,24^ The here presented approach is based on wavelength flexible conversion to the mid-infrared (MIR) via difference frequency generation (DFG) between a widely tunable NIR optical-parametric oscillator (OPO) and an electro-optic dual frequency comb at 1550 nm wavelength. The wavelength flexible conversion into the MIR compensates for the moderate spectral coverage of the dual comb. The main goal of this work is to investigate how the dual comb spectrometer can perform this demanding measurement task. The achievable precision was investigated as well as the linearity of the spectrometer for the determination of the stable isotopic ratio of CO_2_. Although there are commercially available systems allowing fast, precise and accurate optical measurements of the stable isotopic ratio of CO_2_, there is to our knowledge no laser-based system capable of performing precise measurements under ambient pressure.

## Experimental Setup and Methods

### Spectrometer Description

The dual comb spectrometer used in this study was developed by Nitzsche et al.^25^ The complete schematic of the setup is depicted in Figure 1. The two frequency combs are generated via fast electro-optic intensity modulation of a common continuous wave (cw) fiber laser at 1550 nm and subsequent spectral broadening in two 10 km long dispersion-compensating fibers (DCF). This results in a flexible dual comb regarding spectral resolution, ranging from 0.004 cm^–1^ to 0.016 cm^–1^ (130 to 500 MHz) and spectral coverage which can be adjusted from 5 to 20 cm^–1^ (150 to 600 GHz). For the measurement of the stable isotopic ratio of CO_2_ a spectral resolution of 0.004 cm^–1^ (130 MHz) and a spectral coverage of 8 cm^–1^ was chosen, where several configurations were tested to reach the highest precision. This results in an average signal-to-noise ratio per comb mode of 51 dB and a noise equivalent absorption coefficient (NEA) of 5.4(9)·10^–6^ cm^–1^ Hz^–1/2^, which is described in more detail in the Supporting Information. To address the strong fundamental absorptions of small molecules, like the here targeted CO_2_ around 4.3 μm wavelength (∼2300 cm^–1^), the dual comb is converted to the MIR via difference frequency mixing in a periodically poled LiNbO_3_ (PPLN) crystal with a continuous wave OPO. The continuous tuning range of the OPO of 1 to 1.5 μm wavelength theoretically allows for a flexible positioning of the comb spectrum between 2.8 and 46 μm wavelength depending on the used crystal for difference frequency generation but is currently limited to a wavelength range of 3 to 5 μm (3333 to 2000 cm^–1^), because of the used PPLN. The OPO′s wavelength is continuously measured with a wavelength meter (WS7/High Finesse) with an accuracy of 60 MHz. The measured short-term drift of the OPO is 20 kHz/s. The spectrometer is capable of taking spectra with an acquisition rate of 10 Hz.

**Figure 1:**
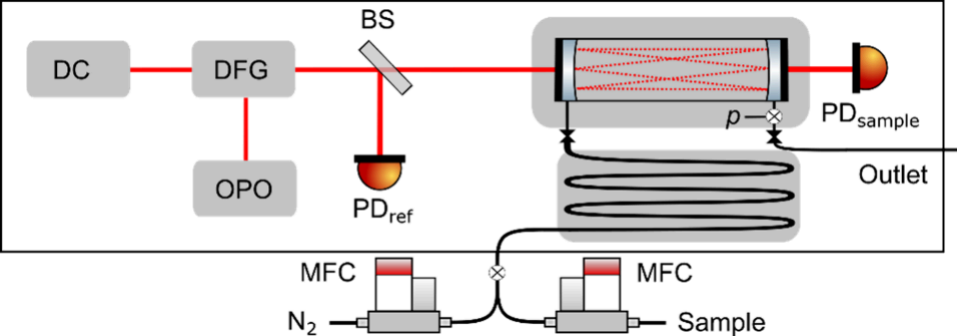
Experimental setup of the dual comb spectrometer. DC: dual comb, DFG: difference frequency generation, OPO: optical-parametric oscillator, BS: pellicle beam splitter, PD_ref_: reference photodetector, PD_sample_: sample photodetector, MFC: mass-flow controller, *p*: pressure sensor.

The generated MIR dual comb with a measured optical power of 0.70(4) mW is split in two branches via a Pellicle Beamsplitter (Thorlabs BP145B4), where one branch directly goes to a reference detector (VIGO PVI-4TE). The other branch is coupled to a gas cell of the Herriott-type (Thorlabs HC10L/M-M02) with an absorption path length of 10.44(2) m. The transmitted light is focused on the sample detector (VIGO PVI-4TE). The cell is placed in and thermally connected to a custom-made aluminum housing and insulated with polystyrene. The housing′s temperature is monitored with calibrated 10 kΩ thermistors and fed back to actively controlled heaters, which stabilize the cell temperature above room temperature to 309.15 K with a stability of ± 400 μK. The gas is preconditioned to the cell temperature using a heat exchanger included in the cell housing, which the gas passes before entering the cell. The gas pressure is monitored at a 10 Hz rate with a pressure sensor (Keller PAA-33X) at the gas outlet, whereas the temperature of the housing is monitored at the same rate with the thermistors. The cell periphery including the dual comb source are housed additionally and flushed with pure nitrogen to avoid background absorption of the CO_2_ in laboratory air. The gas cell is filled with the sample gas and subsequently nitrogen using mass flow controllers (Bronkhorst EL-FLOW Prestige), where the cell is opened and sealed via automatically controlled magnetic valves (Bürkert 7011 A 5/64 FKM) to avoid any leakage while measuring.

### Selection of Spectral Region

Due to the capability of the dual comb spectrometer to place the dual comb between 3 and 5 μm wavelength (3333 to 2000 cm^–1^), we have a high flexibility regarding the spectral region. However, there are certain criteria the chosen spectral area has to meet. We identified the ν_3_ band specifically the spectral range between 2280 and 2300 cm^–1^ as sufficient for our purposes. Here, ^12^CO_2_ and ^13^CO_2_ show similar line strengths. Therefore, Figure 2 shows the line intensities, given by the HITRAN 2020 database,^26,27^ of the three major CO_2_ isotopologues. This is required to have comparable signal-to-noise ratios for each isotopologue to achieve the highest precision in the δ^13^C-measurement. Also, the absorption lines show little overlap to other atmospheric gases like water vapor and there is no saturated absorption in the spectrum with CO_2_ concentrations close to atmospheric conditions to not alter the line profiles by the measurement, hence decreasing the accuracy. Figure 3 illustrate a HITRAN simulation of a transmission spectrum of the three main isotopologues and water vapor at atmospheric pressure, a temperature of 298 K, a total CO_2_ concentration of 420 ppmV, a relative humidity of 40% (13,000 ppmV) and an absorption path length of 10.44 m. The simulation shows that for atmospheric conditions the targeted isotopologues show overlapping absorptions with similar strength and no saturated absorption in the chosen spectral area. However, water vapor, as the main interfering gas in the atmosphere, can influence the measurement with an absorption of up to 1%, which has to be avoided by drying atmospheric gas samples.

**Figure 2:**
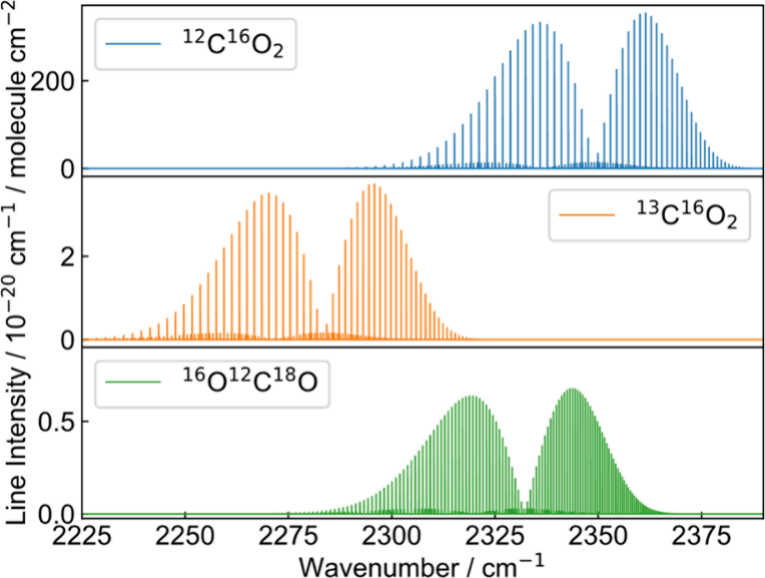
Line intensities of the three most abundant CO_2_ isotopologues ^12^C^16^O_2_, ^13^C^16^O_2_, and ^16^O^12^C^18^O in the ν_3_ band around 2300 cm^–1^ taken from HITRAN.

**Figure 3:**
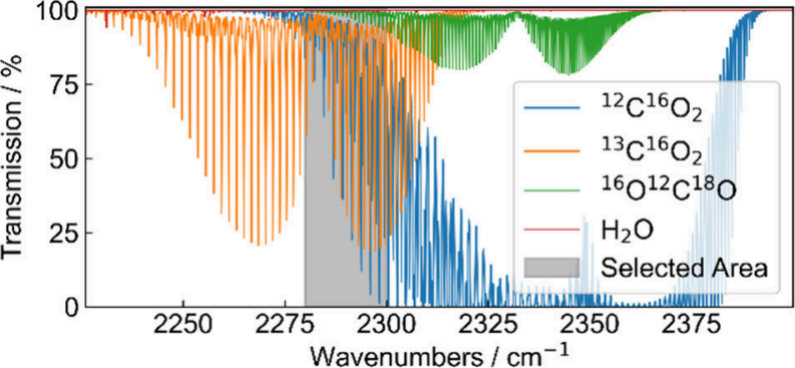
Simulated transmission spectrum of ^12^C^16^O_2_, ^13^C^16^O_2_, and ^16^O^12^C^18^O with a total CO_2_ concentration of 420 ppmV and water vapor (H_2_O) at a concentration of 13,000 ppmV (40% rel. humidity), an absorption path length of 10.44 m, a pressure of 1 atm at a temperature of 298 K. Line parameters and the isotopologue abundances were taken from HITRAN.

To determine the stable isotopic ratio ^13^CO_2_/^12^CO_2_ the δ-notation is used, which puts the measured sample ratio *R*_sample_ in reference to a standard, given with eq 1.

$$\delta^{13}C\left({\mathrm{CO}}_{2}\right)=\left(\frac{R_{\mathrm{sample}}}{R_{\mathrm{VPDB}}}-1\right)\cdot 1000‰$$ 1

Here, *R*_VPDB_ is the Vienna-Pee-Dee-Belemnite standard ratio with a value of 0.0111802.^28^ The transitions of ^12^CO_2_ around 2300 cm^–1^ have higher ground state energies, called hot bands. This means that the precision to determine the stable isotopic ratio is sensitive to temperature fluctuations of the gas sample mainly caused by the measurement of ^12^CO_2_. The theoretical uncertainty of the δ^13^C-value Δδ is according to Bergamaschi et al.^29^ given by

$$\Delta \delta \approx \frac{\Delta E\Delta T}{kT^{2}}$$ 2

where *k* is the Boltzmann constant, *T* the absolute gas temperature with the temperature uncertainty Δ*T* and the difference Δ*E* of the lower state energies between two transitions of ^12^CO_2_ and ^13^CO_2_. Based on eq 2, the needed gas temperature stability for a precision on the δ^13^C-value of Δδ = 0.1‰ is in the order of 40 mK considering the most dominant lines of ^12^CO_2_ and ^13^CO_2_ around 2289 cm^–1^.

### Spectrum Analysis

To retrieve the stable isotopic ratio of atmospheric CO_2_, the concentrations of the isotopologues have to be determined from the measured dual comb spectra. After filling the cell with the sample gas the sample spectrum is taken, consisting of the ratio of the signals of the sample detector *S*_sample_ and the reference detector *S*_ref_. The reference detector is used to account for the complex spectrum of the dual comb resembling a high order Gaussian function, which would complicate spectra fitting. After taking the sample spectrum, the cell is flushed with pure nitrogen to also account for differences in the detection paths, e.g. transmission loss of the gas cell. The resulting measured absorbance spectrum is then given by

$$A=-\ln\left(\frac{{\left(S_{\mathrm{sample}}/S_{\mathrm{ref}}\right)}_{\mathrm{gas}}}{{\left(S_{\mathrm{sample}}/S_{\mathrm{ref}}\right)}_{\mathrm{nitrogen}}}\right)$$ 3

The wavenumber axis of the taken spectra is constructed from the mode spacings of the frequency combs and the central wavelength of the MIR dual comb, which we calculated from the difference frequency of the OPO and the fiber laser. To retrieve the concentrations of the isotopologues from *A*, the spectrum is fitted with a physical model function via a Levenberg–Marquardt least-squares fitting algorithm. The model function is given by

$$f_{\mathrm{model}}=\alpha \left(\mathrm{\nu ̃}-{\mathrm{\nu ̃}}_{0},c_{1},c_{2},c_{3},p,T\right)\cdot L+f_{\mathrm{baseline}}\left(\mathrm{\nu ̃},\mathrm{b⃗}\right)$$ 4

The model function includes the gas-specific absorption coefficient α, which is based on Voigt profiles and spectroscopic parameters given by the HITRAN 2020 database.^26,27^ It depends on the concentrations of ^12^C^16^O_2_, ^13^C^16^O_2_ and ^16^O^12^C^18^O given with *c*_1_, *c*_2_ and *c*_3_, respectively, and includes a global wavenumber shift $\overset{\sim }{\nu }$ _0_. The absorption path length of the gas cell is *L*, where the gas pressure and temperature are *p* and *T* respectively. The baseline function is based on a polynomial of second order given by

$$f_{\mathrm{baseline}}\left(\mathrm{\nu ̃},\mathrm{b⃗}\right)=b_{0}+b_{1}\mathrm{\nu ̃}+b_{2}{\mathrm{\nu ̃}}^{2}$$ 5

where $\overset{\sim }{\nu }$ is the wavenumber. Several polynomial orders have been tested and the second order gave the best results in terms of the root-mean-square error on the residual between the measured spectrum and the fit model. Figure 4 shows an exemplary measured spectrum with 10 s integration time and the simulated contribution of the three considered isotopologues. The spectrum (*S*_sample_) was normalized with a nitrogen spectrum (*S*_nitrogen_), which was taken after the sample measurement with an integration time of 10 s. The residuals between the measured spectrum and the simulation based on the fitted concentrations show structures which indicate deviations of the used line profiles from the measurement profiles. Also, slightly deviating broadening coefficients taken from HITRAN can contribute to the residuals. The strong deviation of the spectrum′s center to the fit is a result of the limited suppression of the carrier frequency by the electro-optic intensity modulators, which does not affect the fit results within their uncertainties.

**Figure 4:**
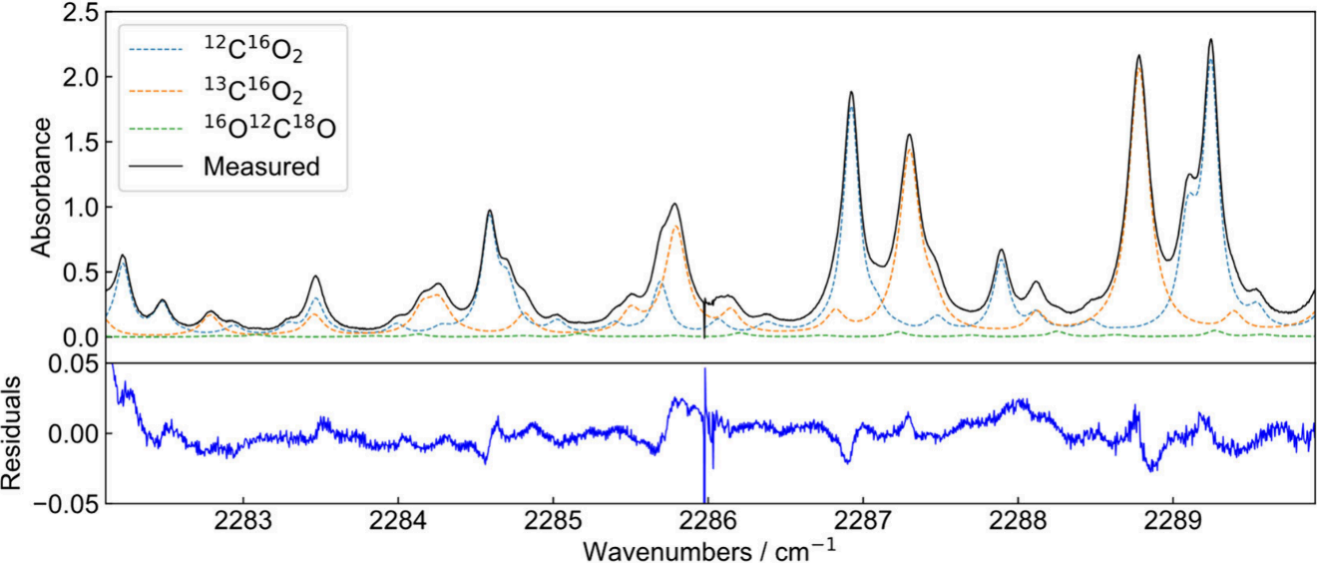
Measured and normalized spectrum with an integration time of 10 s at a CO_2_ concentration of 450 ppm at ambient pressure and a temperature of 309.15 K with simulated contributions of the isotopologues ^12^C^16^O_2_, ^13^C^16^O_2_, and ^16^O^12^C^18^O (upper panel). Residuals between the measured spectrum and the HITRAN simulation (bottom panel).

## Results and Discussion

### Precision of the Spectrometer

To determine the achievable precision in the measurement of the δ^13^C-value in CO_2_, spectra were taken within 2,000 s of acquisition time with a spectra acquisition rate of 10 Hz. The absorption spectra were taken from a calibrated gas sample containing 450(9) ppmV of CO_2_ in dry synthetic air with a calibrated δ^13^C_VPDB_-value of −36.5(5)‰,^28^ where the uncertainty on the δ^13^C-value and the total CO_2_ concentration was given by the standard gas manufacturer. To fit the 20,000 collected spectra, the same pressure was used as the mean value of the 2,000 s measurement, with a measured mean pressure of 993.43(2) mbar. The measured mean temperature of the cell with the thermistors was 309.1499(4) K. The precision for the measurement of the δ^13^C-value of the gas sample was determined by using the Allan deviation, which expresses the behavior of the uncertainty of the measurement over different averaging times τ.^30,31^ For normalization of each of the spectra with 0.1 s integration time (*S*_sample_), only one nitrogen spectrum (*S*_nitrogen_) was used, which was taken after the measurement with an integration time of 10 s. The δ^13^C-values were calculated with eq 1 from the measured concentrations of ^12^CO_2_ and ^13^CO_2_, determined by fitting each normalized spectrum with eq 4 in a first step to correct for the underlying baseline and wavenumber shift. The spectra were then corrected with the found baseline parameters and the wavenumber shift and subsequently the isotopologue concentrations were fitted from the corrected spectra to minimize fitting errors on the concentration values. The relative fitting error for the concentration retrievals of ^12^CO_2_ and ^13^CO_2_ is in the range of 10^–3^, whereas the fitting error for ^16^O^12^C^18^O is 10^–2^ due to the weak absorption. Figure 5 shows the Allan-Werle plots^30^ for the concentrations of ^12^CO_2_ (a) and ^13^CO_2_ (b) for the respective fit results of the 2,000 s measurement and the Allan-Werle plot for the resulting δ^13^C-values (c). For ^12^CO_2_ a precision of 0.08 ppm for 396 s averaging time was achieved, likewise 0.9 ppb for ^13^CO_2_ (corresponding to relative precisions of 1.9·10^–4^). The Allan deviation analysis of the δ^13^C-values gives a precision of <0.1‰ after 172 s. The measurement was conducted in the spectral range of 2282 cm^–1^ to 2290 cm^–1^, where different spectral ranges between 2280 cm^–1^ and 2300 cm^–1^ have been tested for the optimum precision. The dashed lines depict the theoretical behavior of white noise proportional to $1/\sqrt{\tau }$ . The Allan deviation of the δ^13^C-values follows the expected behavior for white noise. At 400 s the Allan deviations of the concentrations start to increase indicating that system drifts influence the measurement. However, for a clear statement about system drift the measurement time is too short and longer measurements have to be carried out in the future. We observe an unexpected behavior for short averaging times, where the Allan deviations plateau until they decrease for integration times longer than 1 s.^32^ This is caused by periodic fluctuations of the fitted concentration values with a rate of 1.4 Hz. To further investigate this behavior, we analyzed the measured pressure and thermistor temperature by Fourier-transforming the measured signals. No periodical fluctuations with a frequency of 1.4 Hz were observed. In a second step we investigated the fitted baseline parameters with the same method. All three fitted baseline parameters show oscillations at a frequency of 1.4 Hz indicating that the dual comb intensity fluctuates at that frequency, hence influencing the determination of the isotolopogue concentrations. This behavior is likely caused by polarization fluctuations in the DCFs, which translates to intensity fluctuations in the dual comb spectrum. The DCFs are non-polarization maintaining fibers, where the polarization is optimized after the DCFs with waveplates before measurements, but not controlled actively. The DFCs are placed in a housing to protect them from mechanical vibrations, but they are still sensitive to temperature fluctuations, which can alter the polarization within the fibers. This can be addressed by adding active polarization control.

**Figure 5:**
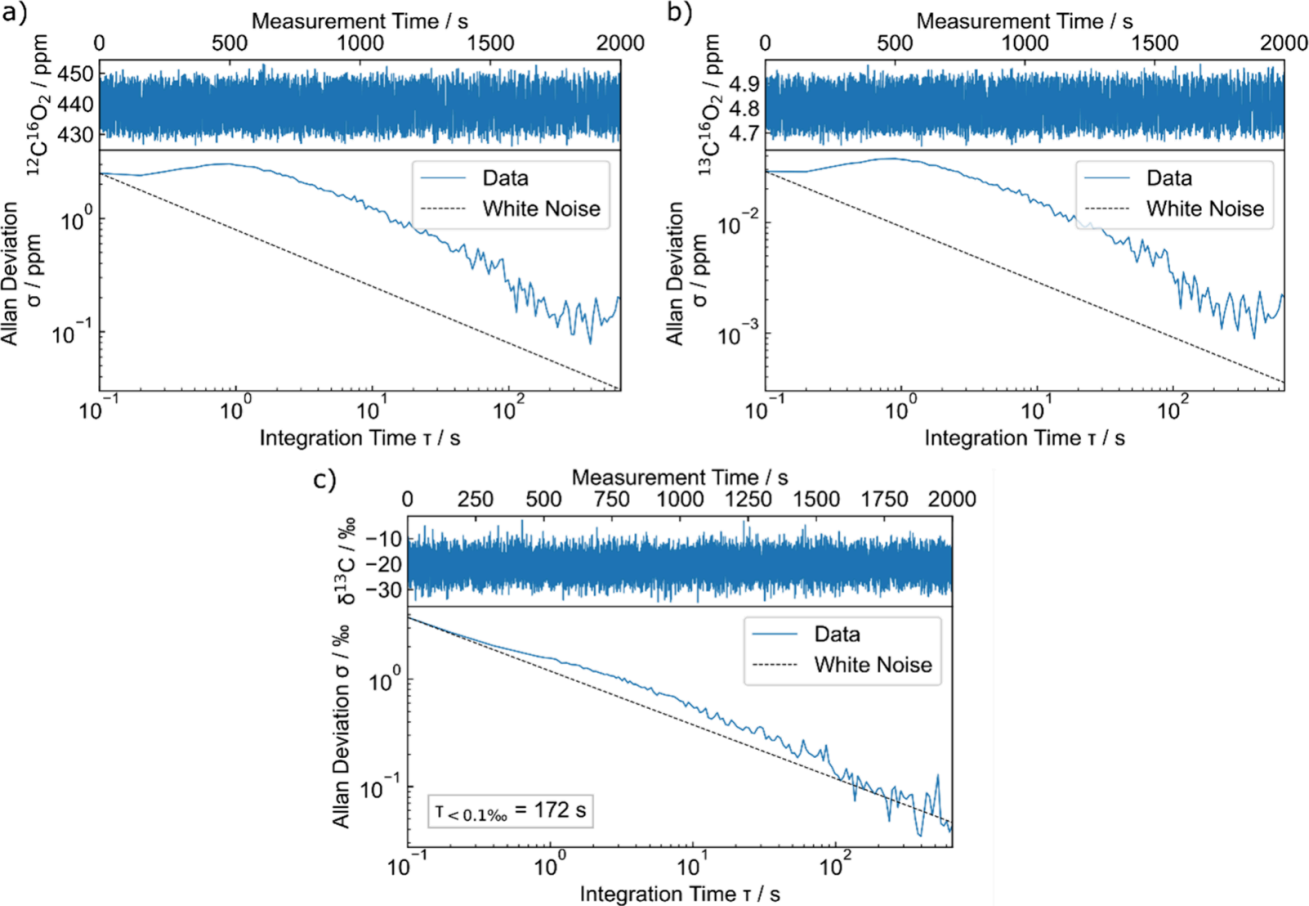
Measurement time series (upper plot panels) and Allan deviations (lower plot panels) for ^12^C^16^O_2_ (a), ^13^C^16^O_2_ (b), and the associated δ^13^C-values (c). The dashed lines show the theoretical behavior for white noise.

### Calibration and Linearity of δ^13^C Measurements

To calibrate the δ^13^C-measurements, the two-point δ^13^C-value calibration method was used by using two standard gases with δ^13^C_VPDB_-values of −36.5‰ and −5.4‰ with a known total CO_2_ concentration of 450(9) ppm. The calibration equation is given by

$$\delta_{s,t}=m\cdot \delta_{s,m}+b$$ 6

where δ_s,t_ and δ_s,m_ are the true and measured sample δ^13^C-values,

$$m=\frac{\delta_{1,t}-\delta_{2,t}}{\delta_{1,m}-\delta_{2,m}}$$ 7

and

$$b=\delta_{1,t}-\frac{\delta_{1,t}-\delta_{2,t}}{\delta_{1,m}-\delta_{2,m}}\delta_{1,m}$$ 8

according to Wen et al.^33^ and Griffith.^34^ δ_i,t_ and δ_i,m_ are the true and measured values of the standard gases 1 and 2, respectively. The linearity of the δ^13^C measurements was investigated by mixing the two standard gases in linear steps. The set points of each mixture are given in Table 1. Per mixture, spectra with an integration time of 200 s were taken to ensure the needed precision and normalized with subsequent nitrogen spectra with an integration time of 10 s. The resulting δ^13^C-values were calibrated using eqs 6 to 8 with δ_1,t_ = −36.5‰ and δ_2,t_ = −5.4‰. Figure 6 shows the calibrated measurement results for each mixture. The uncertainty of the measurements of <0.1‰ for 200 s integration time was derived from Allan deviation analysis. The uncertainty on the set points is mainly given by the accuracy of the mass flow controllers and is <0.01‰. The linearity of the measurement was analyzed using the coefficient of determination *R* ^2^ resulting in *R* ^2^ = 0.998. The result of the mixture with an expected δ^13^C-value of −15.8‰ was excluded from the linearity analysis as the measurement was corrupted by an unexpected jump in the OPO′s wavelength within the measurement time. This also influenced the mixtures for δ^13^ *C* = −20.1‰ and δ^13^ *C* = −18.4‰ where the wavelength drifted. This caused the clear deviation from the linear behavior.

**Figure 6:**
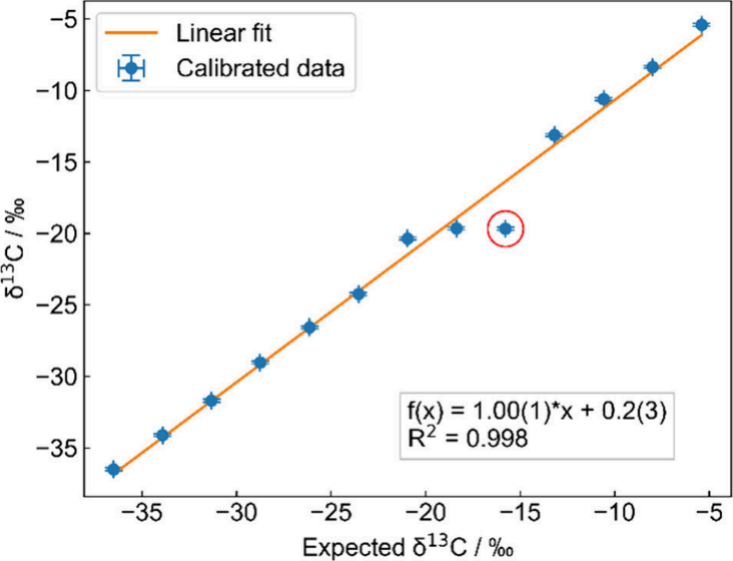
Calibrated δ^13^C measurements for 13 mixtures (including the pure samples) of the two calibrated gas samples with δ^13^C_VPDB,1_ = −36.5‰ and δ^13^C_VPDB,2_ = −5.4‰ for an integration time of 200 s. The linear fit shows a coefficient of determination *R* ^2^ = 0.998. The red circle marks the data point, which was disregarded for the linearity analysis.

**Table 1:** Set Mixtures of Two Calibrated Standard Gases with the Same Total CO_2_ Concentration of 450 ppm and Their Corresponding δ^13^C Values with an Uncertainty of <0.01‰

Mixture	1	2	3	4	5	6	7	8	9	10	11	12	13
δ^13^C (‰)	–36.5	–33.9	–31.3	–28.8	–26.1	–23.5	–21.0	–18.4	–15.8	–13.2	–10.6	–8.0	–5.4

### Concentration Dependency of δ^13^C Measurements

To investigate the behavior of the δ^13^C-values with changing CO_2_ concentration, two standard gases with the same δ^13^C_VPDB_-value of −36.5(5)‰ but with different calibrated total CO_2_ concentrations of 300(6) ppm and 450(9) ppm were mixed in steps of 15 ppm. The uncertainty on the total CO_2_ concentration of each standard gas was given by the manufacturer. Again, per mixture, spectra with an integration time of 200 s were taken and normalized with subsequent nitrogen spectra with integration times of 10 s. The linearity analysis of the concentration values (see top panels of Figure 7) resulted in coefficients of determination of *R* ^2^ = 0.999 for both isotopologues. Due to the high linearity of the isotopologue concentration measurements, the resulting δ^13^C-values were calibrated using the two-point mixing ratio calibration method.^33^ Therefore, the concentrations of the isotopologues were calibrated with equation

$$c_{s,t}^{n}=\frac{c_{2,t}^{n}-c_{1,t}^{n}}{c_{2,m}^{n}-c_{1,m}^{n}}\left(c_{s,m}^{n}-c_{1,m}^{n}\right)+c_{1,t}^{n}$$ 9

where the superscript *n* stands for the isotopologues ^12^CO_2_ and ^13^CO_2_ respectively and *c*_i,t_ and *c*_i,m_ are the true and measured concentrations of the standard gases 1 and 2 respectively with the measured concentration of the sample gas *c*_s,m_. The total CO_2_ concentration is given by

$$c_{\mathrm{total}}=c_{12}+c_{13}+f\cdot c_{\mathrm{total}}$$ 10

**Figure 7:**
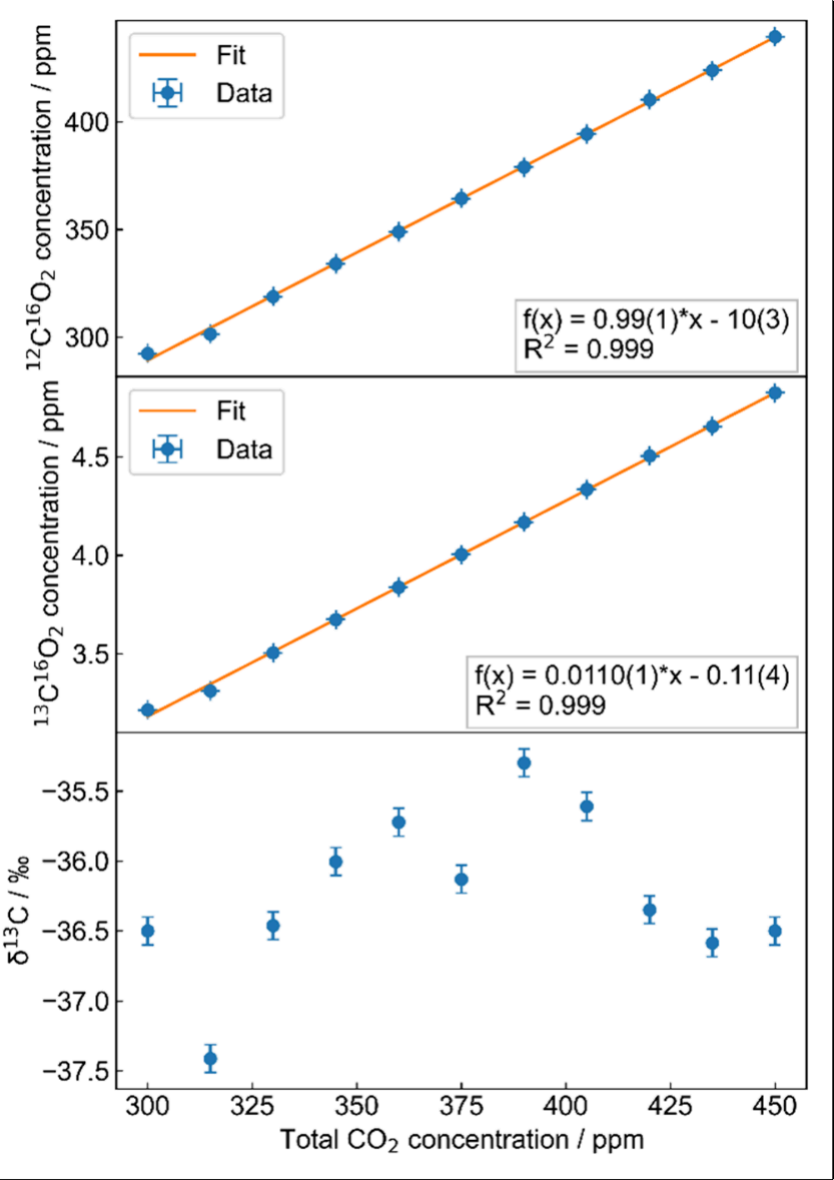
Measured concentrations of ^12^CO_2_ (top panel), ^13^CO_2_ (middle panel) as a function of total CO_2_ concentration, and the resulting calibrated δ^13^C-values (bottom panel) for total CO_2_ concentrations ranging from 300 to 450 ppm. The uncertainty on the set points is given by the accuracy of the used mass flow controllers.

Here, the fraction of all isotopologues, which are not ^12^CO_2_ or ^13^CO_2_ is *f* = 0.00474. The true concentration of ^12^CO_2_ was calculated with

$$c_{12}=\frac{c_{\mathrm{total}}\left(1-f\right)}{1+(R_{\mathrm{VPDB}}\left(1+\frac{\delta_{\mathrm{standard}}}{1000‰})\right)}$$ 11

with the isotopic ratio of the Vienna-Pee-Dee-Belemnite standard *R*_VPDB_ and the known δ^13^C-value of the standard gases. From equations 10 and 11, the concentration of ^13^CO_2_ can be calculated. The resulting calibrated concentrations for the isotopologues ^12^CO_2_, ^13^CO_2_ (top and middle panel) and the calibrated δ^13^C-values (bottom panel) are shown in Figure 7. Calibration of the isotopologue concentrations with equations 9 to 11 should eliminate the dependency of the δ^13^C-value to the total CO_2_ concentration.^33^ Although no systematic dependence on the total CO_2_ concentration is observed, the measurements show deviations from the expected behavior. These deviations can be caused by inaccurate physical fitting models because of line broadening effects due to the various gases present in air, sensitivity of the spectra to temperature and pressure fluctuations or deviations of the line profiles used in the physical model to the “real” profiles. For future investigations the gas cell will be modified to measure the gas temperature directly within the cell. This will give insight on temperature variations between each measurement and how they contribute to the observed deviations.

## Conclusion and Outlook

In this work, we demonstrated the capability of our dual comb spectrometer to precisely measure stable isotopic ratios of CO_2_ under ambient pressure. To our knowledge, this is the first laser-based system to perform measurements with a precision of <0.1‰ under these conditions. The spectrometer is based on an electro-optic dual comb, which is generated in the near-infrared at a wavelength of 1550 nm and subsequently frequency converted to the mid-infrared to reach the strong fundamental ν_3_ absorption band of CO_2_ at around 4.3 μm wavelength. The spectrometer offers a high signal-to-noise ratio per comb mode of around 51 dB with a noise equivalent absorption coefficient of 5.4(9)·10^–6^ cm^–1^ Hz^–1/2^ at a spectral resolution of 130 MHz and spectral coverage of 8 cm^–1^. This provides a simultaneous measurement of the concentrations of the isotopologues ^12^C^16^O_2_, ^13^C^16^O_2_ and ^16^O^12^C^18^O. The broad spectral coverage compared to tunable lasers allows for stable isotopic ratio measurements of the two main isotopologues ^12^CO_2_ and ^13^CO_2_ (δ^13^C) under ambient pressure with high precision. For integration times above 172 s a precision of <0.1‰ was achieved. Linearity analysis of the measured δ^13^C-values showed a high linearity with a coefficient of determination *R* ^2^ of 0.998 of gas samples with varying δ^13^C-values typically found in the atmosphere according to the National Oceanic and Atmospheric Administration.^35^ The linearity analysis for varying total CO_2_ concentrations ranging from 300 to 450 ppm revealed coefficients of determination of 0.999 for each isotopologue. These results indicate that dual comb spectroscopy is a feasible alternative to classical tunable laser absorption spectroscopy for the analysis of stable isotopic ratios of CO_2_ especially for measurements under atmospheric pressure.

Further improvements to the spectrometer include the implementation of direct measurement of the gas temperature within the gas cell and gas driers. This will enable field deployment to analyze samples of ambient air because water vapor can influence the absorption profiles of the investigated gases. Additionally, active control of the OPO′s wavelength to a fixed reference wavelength will be implemented to further decrease system drift and increase precision.

## Supplementary Material



## Notes

Methods used to determine the comb mode signal-to-noise ratio and the noise equivalent absorption coefficient of the spectrometer (PDF)
