# Supplementary material for: Dual Comb Spectrometer for the Determination of Stable Isotopic Ratios of Atmospheric CO2 with Sub-Permille Precision at Atmospheric Pressure
Source: Anal Chem. 2025 Dec 16;97(51):28254–61. doi: 10.1021/acs.analchem.5c04845 (PMC12756849; doi:10.1021/acs.analchem.5c04845)
Supplement: Supplementary file 1 [file ac5c04845_si_001.pdf]

Dual comb spectrometer for the determination of stable isotopic ratios of atmospheric CO_2 with sub-permille precision at atmospheric pressure

Jens Goldschmidt,[1,*] Nicolas Brugger,[1] Leonard Nitzsche,[1] Ponkanok Nitzsche,[1] Cem Dinc,[1] Christian Weber,[1,2] Ingo Breunig,[3] Katrin Schmitt,[1,2] Frank Kühnemann[2,4] and Jürgen Wöllenstein[1,2]

[1]Laboratory for Gassensors, Department of Microsystems Engineering – IMTEK, University of Freiburg, Georges-Köhler-Allee 102, 79110 Freiburg, Germany

[2]Fraunhofer Institute for Physical Measurement Techniques – IPM, Georges-Köhler-Allee 301, 79110 Freiburg, Germany

[3]Laboratory for Optical Systems, Department of Microsystems Engineering – IMTEK, University of Freiburg, Georges-Köhler-Allee 102, 79110 Freiburg, Germany

[4]Institute of Physics, University of Freiburg, Hermann-Herder-Straße 3, 79104 Freiburg, Germany

KEYWORDS. Dual Comb Spectroscopy, Frequency Comb Spectroscopy, Laser Spectroscopy, Stable Isotopic Ratio of CO_2, Trace Gas Analysis

*Corresponding author: jens.goldschmidt@imtek.uni-freiburg.de

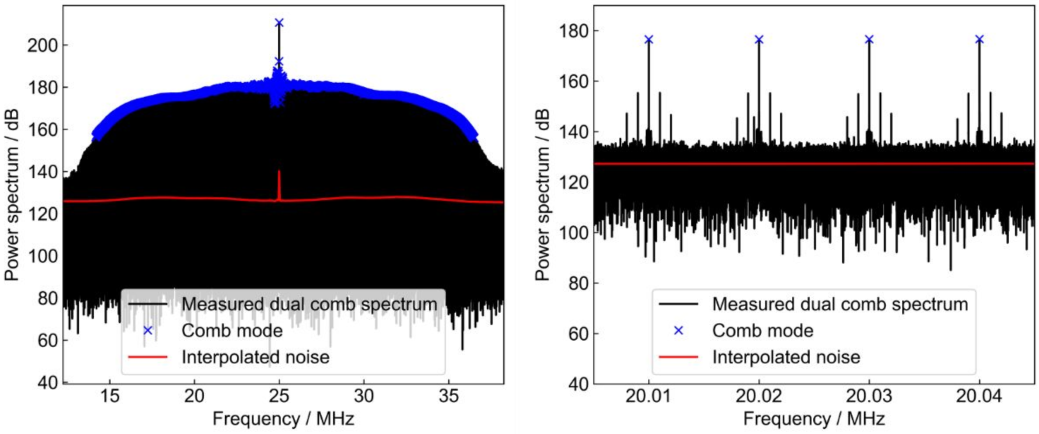

S1. Measured power spectral density of the dual comb in the radio frequency domain with comb repetition rates of 130 MHz and 129.99 MHz (left panel). Zoom into the spectrum at a radio frequency of 20 MHz with four fully resolved comb modes (right panel). The interpolated noise is calculated in between and 2.2 kHz away from the respective comb modes as the mean value of the power spectral density.

Signal-to-noise ratio of the dual comb

To determine the signal-to-noise ratio (SNR) of the dual comb the measured radio frequency (RF) spectrum is analyzed. Therefore, an interferogram for 1 s measurement time is taken, which results from the beating between the two combs with repetition rates of 130 MHz and 129.99 MHz. The power spectral density (PSD), i.e. the dual comb spectrum, in the RF domain is obtained by a Fast Fourier Transformation (FFT) giving a 1 Hz frequency resolution. The measured RF spectrum of the full dual comb is depicted in S1 (left panel). Because every frequency in the system is precisely known due to referencing them to a single GPS synchronized 10 MHz clock, we precisely know the spectral position of every comb mode. The mode spacing is 10 kHz, according to the difference in repetition rates of the two combs. For the SNR determination we interpolate the noise floor in the spectrum by taking the mean value of the PSD in between the comb modes and 2.2 kHz away from the respective modes. For clarification the right panel of S1 shows a zoomed-in spectrum for four

comb modes around 20 MHz. The SNR was then calculated by taking the mean difference of the amplitudes of the selected comb modes and the interpolated noise floor, where only the comb modes 30 dB above the noise floor are chosen to calculate the SNR. The analysis of the spectrum resulted in an average SNR per comb mode of 51 dB. The peak in the PSD in the left panel of S1 is a result of the finite extinction ratio of the used intensity modulators (Modbox-PG-CBand-50 / iXBlue), where unmodulated light of the seed-laser is still present. The dominant sidebands around the comb modes (right panel in S1), located 1 kHz away from the comb modes, are a result of the active modulation to stabilize the intensity modulators to generate the combs. Other visible sidebands are accounted for harmonics of the stabilization frequency, as well as mechanical noise. The mechanical noise can couple to the dispersion compensating fibers, which were used to spectrally broaden the combs. This results in a polarization modulation within the fibers and in combination with the polarization controllers in intensity modulations[1].

Noise equivalent absorption coefficient

To determine the noise equivalent absorption coefficient (NEA), we take the standard deviation of the normalized absorbance spectrum, where no gas absorption is present. The spectrum was retrieved by a measurement of a sample spectrum with 1 s of integration time and a subsequent reference spectrum with 1 s of integration time to normalize the sample spectrum. Additionally, we fitted a polynomial function of second order to the spectrum and subtracted it from the spectrum to get a flat baseline. By taking the absorption path length of the spectrometer´s cell of 10.44 m into account this resulted in an average NEA of $5.4(9)\cdot10^{-6}$ cm^{-1} $Hz^{-1/2}$ for six measurements with an accumulated integration time of 2 s per measurement[1].

[1]Nitzsche, L.; Goldschmidt, J.; Kiessling, J.; Wolf, S.; Kühnemann, F.; Wöllenstein, J. Tunable dual-comb spectrometer for mid-infrared trace gas analysis from 3 to 4.7 µm. *Optics express* **2021**, *29*, 25449–25461.
